# A novel low-complexity digital filter design for wearable ECG devices

**DOI:** 10.1371/journal.pone.0175139

**Published:** 2017-04-06

**Authors:** Shadnaz Asgari, Alireza Mehrnia

**Affiliations:** 1 Biomedical Engineering Department, California State University, Long Beach, California, United States of America; 2 Computer Engineering and Computer Science Department, California State University, Long Beach, California, United States of America; 3 Electrical Engineering Department, University of California Los Angeles, Los Angeles, California, United States of America; University of Illinois at Urbana-Champaign, UNITED STATES

## Abstract

Wearable and implantable Electrocardiograph (ECG) devices are becoming prevailing tools for continuous real-time personal health monitoring. The ECG signal can be contaminated by various types of noise and artifacts (e.g., powerline interference, baseline wandering) that must be removed or suppressed for accurate ECG signal processing. Limited device size, power consumption and cost are critical issues that need to be carefully considered when designing any portable health monitoring device, including a battery-powered ECG device. This work presents a novel low-complexity noise suppression reconfigurable finite impulse response (FIR) filter structure for wearable ECG and heart monitoring devices. The design relies on a recently introduced optimally-factored FIR filter method. The new filter structure and several of its useful features are presented in detail. We also studied the hardware complexity of the proposed structure and compared it with the state-of-the-art. The results showed that the new ECG filter has a lower hardware complexity relative to the state-of-the-art ECG filters.

## Introduction

Cardiovascular disease is the leading cause of death worldwide, accounting for 30% of global mortalities [1]. In 2010, the estimated total cost of cardiovascular disease in the United States alone was $444 billion [2]. With the demographic shift toward an older population, these costs are expected to increase substantially over the next decade [3]. Continuous monitoring of the heart and its functionality enhances the early diagnosis, intervention, or prevention of cardiovascular diseases [4].

Recent advances in wireless communications and integrated circuits can considerably improve the healthcare management and cost [5]. For example, utilizing smart wearable electrocardiogram (ECG) devices can significantly enhance healthcare of the patients with cardiovascular diseases. Although much effort has been put into the development of such devices over the past few years, integration of these technologies into the clinical practice still remains limited, mainly due to reliability and practicality issues [5].

**Fig 1:**
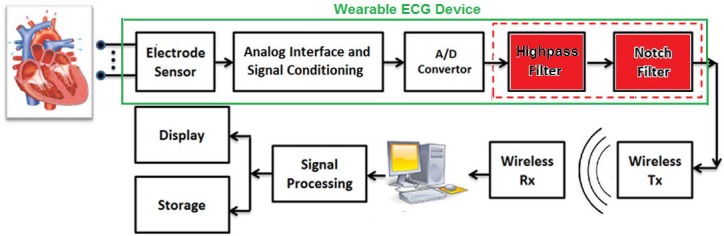
A schematic presentation of a wearable ECG device where the captured signal is transmitted (through a wireless channel) to a remote backend system for further processing.

Several requirements (in terms of power consumption, physical size, and cost) must be taken into consideration to design a reliable and practical wearable ECG device. For example, a lower power consumption would guarantee a longer battery life. Hardware complexity of the device would also affect the weight, size and cost of the device. As depicted in Fig 1, a wearable ECG device consists of several components in addition to the ECG electrodes (e.g., Analog interface and signal conditioning, A/D converter). The hardware complexity of each of these building blocks would affect the power consumption, size and cost of the device. One of the main components of a wearable ECG device is the filtering block. ECG signal is often contaminated by various types of noise and artifacts that need to be removed and/or suppressed by employing hardware efficient filters [6].

In general, ECG signal contaminants can be classified into six categories [7–11]:

- Powerline interference is a narrow-band noise centered at 60 Hz or 50 Hz (depending on the country of operation) and its harmonics, with a bandwidth of less than 1 Hz [8]. The analog front-end processing circuitry consists of an instrumentation amplifier and a filter that can reduce the powerline interference. Nonetheless, ECG recordings are often contaminated by residual powerline interference due to differences in electrode impedances and also because of stray currents through the patient and the cables [9].
- The electrode contact noise is due to electrode “popping” or a loose contact with the skin.
- Motion artifacts are generated by changes in the skin-electrode impedance because of a patient’s movement during recording.
- Instrumentation noise is due to radio frequency interference from other equipment, e.g., implanted devices such as pacemakers.
- The baseline drifts are mainly caused by respiration and perspiration.
- Electromyographic (EMG) noise is induced by electrical activities of skeletal muscles during periods of contraction.

The EMG noise tends to be non-stationary and has a frequency range that overlaps that of the original ECG signal (spanning from slightly below 1 Hz up to 120 Hz). This type of noise cannot easily be removed by hardware, and hence it is usually corrected in software. The powerline noise suppression can be achieved by applying a notch filter with extremely sharp transition bands around each notched frequency. Majority of other ECG noises (i.e., electrode contact noise, motion artifacts and baseline drifts) have a frequency content below 0.5 Hz. Therefore, a high-pass filter should be carefully designed to remove these noises without distorting the important clinical information hidden in the low frequency content of ECG signal (e.g., signal segment between S and T components of ECG).

Several approaches have been proposed for ECG noise suppression [11]. One approach eliminates noise at the analog front-end [12]. It is well known that such analog filtering suffers from nonlinear phase response and a difficulty in making very deep attenuations (notches) at precise frequencies [13]. Furthermore, analog filters can have high sensitivity to component aging and variations over time—both unavoidable issues in long-term heart-monitoring with wearable ECG devices. Another approach is to eliminate ECG noise and contaminants (at the software level) using complex ECG noise-cancellation algorithms [9]. However, these algorithms usually have high computational burden, and therefore are not appropriate for hardware implementation.

Given the limitations of these two methods, a practical alternative to eliminate noise and artifacts in real-time is the use of a digital filter with the following specifications: deep notching of the noise at direct current (DC), 50/60 Hz, and 100/120 Hz without adversely affecting the ECG signal content by limiting the passband ripple to ±0.25 dB or ±0.5 dB (depending on the application). Such deep notching requires at least 40-dB attenuation (and up to 60-dB in some applications) at the aforementioned frequencies with extremely sharp transition bands that must be within approximately ±0.5 Hz from each notched frequency [8–11]. Note that with this approach, if needed, analog filtering can still be used for basic preliminary removal of high-frequency noise prior to an analog-to-digital converter.

Over the last few decades, several research groups have employed different techniques to design the above digital filter. William et al. used infinite impulse response filter to meet the notch-filter specifications [14]. However, this approach suffers from nonlinear phase response distortion.

**Fig 2:**
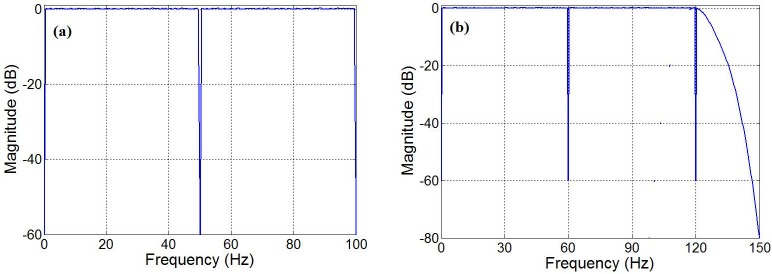
Illustration of target filter for two scenarios: (a) Scenarios I described in [13] with a sampling frequency of 200 Hz, (b) Scenario II described in [10] with a sampling frequency of 300 Hz.

On the other hand, the authors in [13,15] have employed recursive running sum finite impulse response (FIR) digital filters [16,17] for the DC and 50-Hz notch filters with sampling rates of 200 Hz, as shown in Fig 2(A) (Throughout the paper, we shall call this Scenario I). Furthermore, the authors in [10] and [18] have employed the frequency response masking method [19] to notch DC, 60 Hz, 120 Hz and above for a 300-Hz sampling rate scenario, as shown in Fig 2(B) (We shall refer to this as Scenario II).

In general, the use of linear phase FIR filters for wearable ECG devices have several advantages including minimum signal distortion and low cost [10]. However, linear phase FIR filters usually lack the desired hardware efficiency due to their high complexity.

In this paper, we propose a low-complexity linear phase FIR filter design for the ECG noise suppression. Our method employs a newly-developed optimally factored filter technique [20–22] combined with the optimally-stretched interpolated FIR method [23] to create a low complexity cascaded filter structure. Two design scenarios of Fig 2 are discussed in details and their hardware complexities are compared with the published state-of-the-art.

## Methods

Studies have shown that the hardware complexity of linear phase FIR filters could be reduced by realizing the filter using a cascade of a scaled sequence of stages, each representing a factor of the FIR filter's transfer function [20]. A method is recently developed to find the best FIR filter factors [21]. In addition, this method is able to carefully scale the factors and sequence them, such that the resulted cascaded structure has the minimum hardware complexity [22]. Here we use the method to design a hardware efficient ECG filter. In the following subsections, we describe our design in details for two different scenarios.

### Proposed filter design for Scenario I

In Scenario I, as depicted in Fig 2(A), the target filter *H*(*z*) must satisfy the following requirements for the sampling rate of 200Hz:

- 60 dB notches at DC, 50 Hz (equivalent to 0.5π) and 100 Hz (equivalent to π).
- passband frequency ranges of (0.5 Hz- 49.5 Hz) and (50.5 Hz- 99.5 Hz).
- passband ripple (*δ*_*p*_) of 0.5 dB peak-to-peak.

**Fig 3:**
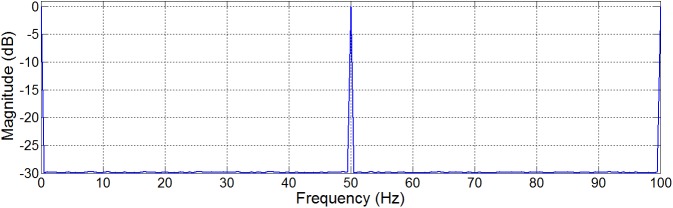
Magnitude response of the complement filter *U*(*z*) for Scenario I.

For the efficient design of *H*(*z*), we initially focus on realizing its “complement filter” *U*(*z*) defined as

$$U(z)=z^{-N/2}-H(z),$$ (1)

where *N* is the order of the *H*(*z*) filter. Given the target magnitude response of *H*(*z*), the complement filter *U*(*z*) has a magnitude response shown in Fig 3. Note that the filter *U*(*z*) enables the use of interpolated FIR (IFIR) technique [19, 23] to reduce hardware complexity (as shall be shown later).

The target passband ripple (*δ*_*P*_) of 0.5 dB peak-to-peak for *H*(*z*) defines the value of the stopband attenuation for the complement filter *U*(*z*) as the following

$$\text{Stopband}\;\text{of}\;U(z)\leq 20\log_{10}(10^{\delta_{P}/20}-1)\overset{\delta_{P}=0.5\text{dB}}{\Rightarrow }\text{Stopband}\;\text{attenuation}\geq 30.7\text{dB}.$$ (2)

For simplicity, we now focus on the design of a portion of filter *U*(*z*) that only contains its first passband at DC, and then later describe a technique to realize the complete *U*(*z*) filter. We shall refer to this partial version as *U*_Partial_(*z*).

**Fig 4:**
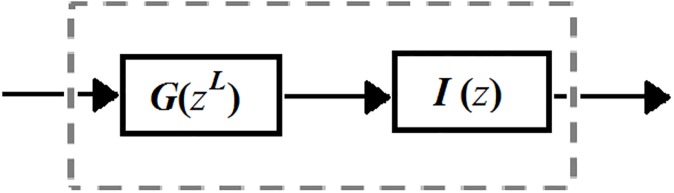
IFIR filter technique employs a cascade of two filters to reduce the number of multipliers and structural adders.

Note that *U*_Partial_(*z*) has a very sharp transition band. In general, IFIR filter techniques are well-suited for the efficient implementation of filters with sharp transition band [19]. The IFIR implementation reduces the filter’s hardware complexity in terms of number of multipliers and structural adders by restructuring it as a cascade of two other filters, a model filter *G*(*z*) and an interpolator (or masking) filter *I*(*z*), as depicted in Fig 4. The parameter *L* is the stretch factor of the IFIR filter implementation which is usually chosen empirically.

An analytical approach is proposed in [23] to determine the optimum choice of the stretch factor (L) to design a filter with minimum number of multipliers. Here, we employ this technique and obtain the optimum value of L for the IFIR implementation of *U*_Partial_(*z*) filter. A hardware complexity study will be conducted later to show the superiority of our IFIR implementation to the conventional Parks-McClellan mini-max FIR realization [24,25] which is based on the Remez exchange algorithm [26].

Following the designs of the model filter and interpolator filter for *U*_Partial_(*z*), we compare their magnitude responses with that of the target filter *U*(*z*), and accordingly modify the interpolator filter *I*(*z*) into *I*_*2*_(*z*) to include the additional passbands at 50Hz and 100 Hz.

Finally, to achieve further reduction in hardware complexity, we use the *factoring* filter design method of [20–22] to decompose the *G*(*z*) and *I*_*2*_(*z*) filters into an optimal sequence of factors (stages) and create an optimally factored-cascade IFIR implementation of the target filter.

To test the performance of the proposed filter design, a series of comprehensive tests are conducted. First, several simulated input signals (e.g., a DC signal, sinusoidal signals at the edge of the filter passband, at 50 Hz and at 100Hz) are fed into our designed filter and their corresponding output signals are studied. We also evaluated the performance of the proposed filter design when the input is field-collected ECG signals contaminated with high levels of DC noise along with 50-Hz and/or 100-Hz powerline sinusoidal noises.

Note that although the proposed filter is designed to remove the narrowband noise at DC, 50 Hz and 100 Hz for the case of a 200-Hz sampling rate, the design can be equally used to remove the noise at DC, 60 Hz and 120 Hz for an ECG signal with sampling rate of 240 Hz.

### Proposed filter design for Scenario II

In Scenario II, as depicted in Fig 2(B), the target filter *H*(*z*) has the following specifications for the sampling rate of 300Hz:

- 60 dB notches at DC, 60 Hz (equivalent to 0.4π) and 120 Hz (equivalent to 0.8π).
- passband frequency ranges of (0.5 Hz- 59.5 Hz) and (60.5 Hz- 119.5 Hz).
- passband ripple (*δ*_*p*_) of 0.5 dB peak-to-peak.
- attenuation of 60 dB at 150 Hz (equivalent to π)

**Fig 5:**
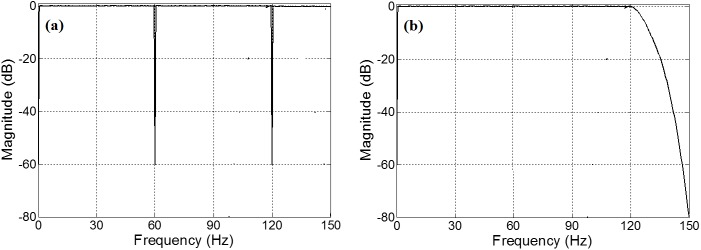
Magnitude responses of the sub-filters for Scenario II-Scheme A: (a) sub-filter *H*_*1*_(*z*); (b) sub-filter *H*_*2*_(*z*).

The signal of interest is contained within the frequency range of 0.5 Hz to 119.5 Hz, while excluding the (59.5 Hz to 60.5 Hz) interval. Given the specification of the target filter *H*(*z*), depicted in Fig 2(B), we can realize it as a cascade of two sub-filters *H*_*1*_(*z*) and *H*_*2*_(*z*) with magnitude responses shown in Fig 5.

Then in order to use IFIR technique for the design of each sub-filter, we focus on realizing their complements *U*_*1*_(*z*) and *U*_*2*_(*z*), where *U*_*1*_(*z*) has narrow passbands at DC, 60 Hz (equivalent to 0.4π), and 120 Hz (equivalent to 0.8π), and *U*_*2*_(*z*) is a high-pass filter from 120 Hz (0.8π) to 150 Hz (equivalent to π). Note that in this scenario, the target filter *H*(*z*) could be later obtained as

$$H(z)=H_{1}(z)H_{2}(z)=(z^{-N_{1}/2}-U_{1}(z))(z^{-N_{2}/2}-U_{2}(z)),$$ (3)

where *N*_*1*_ and *N*_*2*_ are the orders of the sub-filters *H*_*1*_(*z*) and *H*_*2*_(*z*), respectively.

Now to design *U*_*1*_(*z*), similar to Scenario I, we first focus on IFIR realization of *U*_Partial_(*z*) only containing the first passband at DC, by obtaining its model filter *G*(*z*) and interpolator filter *I*(*z*). Then we modify the obtained interpolator filter *I*(*z*) into *I*_*3*_(*z*) to include the additional passbands at 60Hz and 120 Hz.

To design the high-pass filter *U*_*2*_(*z*), we first obtain its low-pass version filter *V*(*z*) over the frequency interval of 0 to 0.2π. Then we decompose this low-pass filter into its IFIR components as *V*(*z*) = *G*_*V*_(*z*)*I*_*V*_(*z*). Note that the low-pass filter *V*(*z*) can be transformed back to the high-pass *U*_*2*_(*z*) by negating its argument, i.e., *U*_*2*_(*z*) = *V*(-*z*).

Finally, the *factoring* filter design method of [20–22] is employed to further decompose each component *G*(*z*), *I*_*3*_(*z*), *G*_*V*_(*z*), and *I*_*V*_(*z*) into an optimal sequence of their factors and create an optimally factored-cascade IFIR implementation of the target filter *H*(*z*).

Note that the proposed filter designs can be equally used to remove the noise at DC, 50 Hz and 100 Hz for an ECG signal with sampling rate of 250 Hz.

## Results

### Filter design for Scenario I

**Fig 6:**
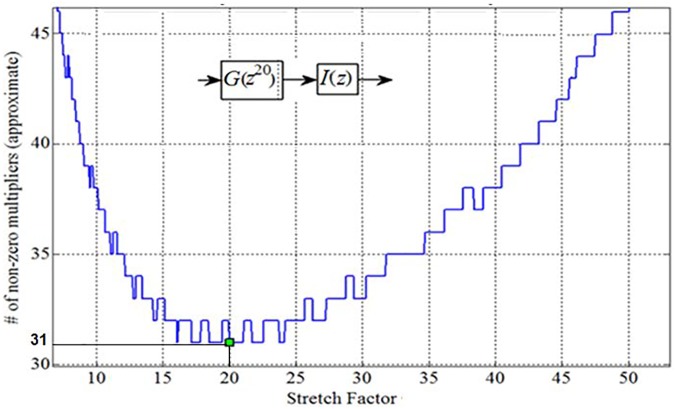
Optimum Stretch Factor for IFIR implementation of *U*_Partial_(z).

Our analysis revealed that the optimum value of the stretch factor L for the IFIR implementation of *U*_Partial_(*z*) is 20. As Fig 6 depicts, the choice of L = 20 minimizes the approximate total number of multipliers to 31.

**Fig 7:**
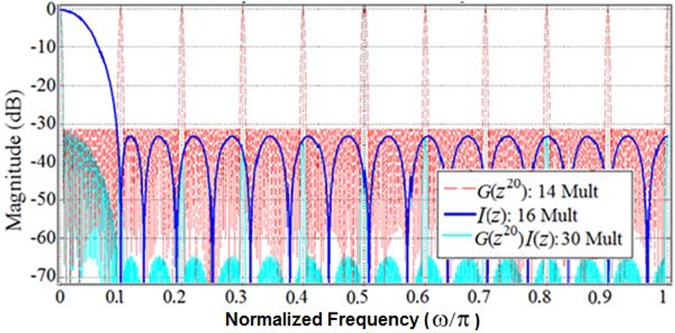
Magnitude responses (dB) of IFIR components of *U*_Partial_(z): *G*(z^20^) and *I*(z).

In fact, using the stretch factor value of 20, we were able to realize the model filter *G*(*z*) with an order of 27 (14 multipliers, 27 structural adders), and the interpolator filter *I*(*z*) with an order of 30 (16 multipliers, 30 structural adders). Fig 7 shows the magnitude responses of the corresponding optimally-stretched IFIR components, *G*(*z*^20^) and *I*(*z*).

Given the orders of the obtained filters *G*(*z*) and *I*(*z*), the effective total order of *U*_Partial_(*z*) = *G*(*z*^20^) *I*(*z*) filter is 20×27+30 = 570. Hence, with our IFIR implementation using the optimal stretch factor, the filter *U*_Partial_(*z*) has an order of 570 and consists of 30 multipliers and 57 structural adders.

**Fig 8:**
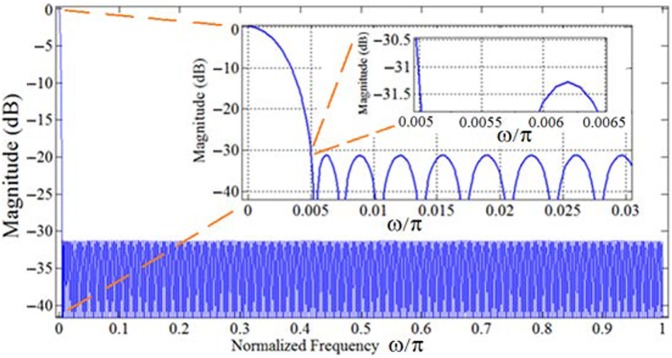
Magnitude response (dB) of the conventional implementation of *U*_Partial_(z).

Fig 8. depicts the magnitude response of the conventional FIR implementation of *U*_Partial_(*z*) using Parks-McClellan method [24,25]. The obtained filter has an order of 547, and consists of 274 multipliers and 547 structural adders.

Hence, the IFIR design reduces the number of multipliers by a factor of nine (274 versus 30 multipliers) and the number of structural adders by a factor of ten (574 versus 57 structural adders), in return for a slight (less than 5%) increase in the total number of delays (registers)—from 547 to 570.

**Fig 9:**
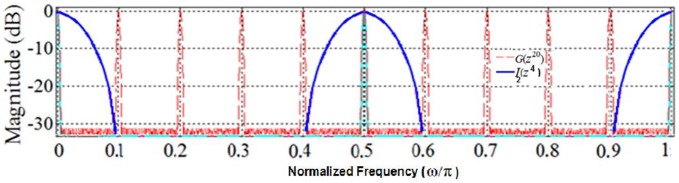
Magnitude responses of IFIR components of *U*(z): *G*(z^20^) and *I*_*2*_(z^4^).

Note that the interpolator filter *I*(*z*) removes all magnitude response peaks except for the first passband at DC as illustrated in Fig 7. On the other hand, the magnitude response of *G*(*z*^20^) provides all the required components of the desired magnitude response of *U*(*z*) at DC, ±0.5π and π. Hence, to convert *U*_Partial_(*z*) to *U* (*z*), we only need to change the interpolator filter *I*(*z*) to avoid the removal of the desired peaks at ±0.5π and π. This desired masking is illustrated in Fig 9 in solid blue which shows the magnitude response of *I*_2_(*z*^4^), the stretched (by a factor of four) version of the new interpolator filter *I*_2_(*z*). In fact, by employing this properly designed interpolator filter, one can select the desired set of peaks from the magnitude response of model filter *G*(*z*^20^). Thus, filter *U*(*z*) is realized using two stretched factors *G*(*z*^20^) and *I*_2_(*z*^4^). Our analysis revealed that the interpolator filter *I*_2_(*z*) has an order of 7 (4 multipliers and 4 structural adders). Hence, the effective total order of *U*(*z*) = *G*(*z*^20^) *I*_2_(*z*^4^) filter is 20×27+4×7 = 568. Therefore, our double-stretch-factor IFIR implementation of *U*(*z*) resulted in a filter with an order of 568, consisting of 18 multipliers and 34 structural adders.

**Fig 10:**
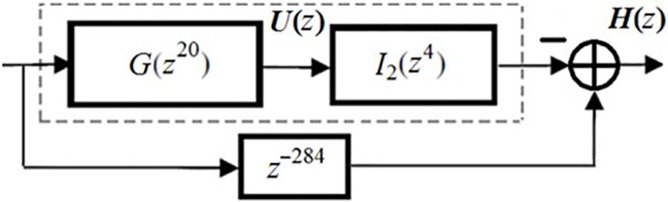
Double-stretch- factor IFIR implementation of *H*(z).

Fig 10 illustrates the double-stretch-factor IFIR implementation of target filter *H*(*z*) obtained from Eq (1), where *N* (the order of the filter) is equal to 568. Note that with our design, the target filter *H*(*z*) has an order of 568 and consists of 18 multipliers and 35 structural adders.

**Fig 11:**
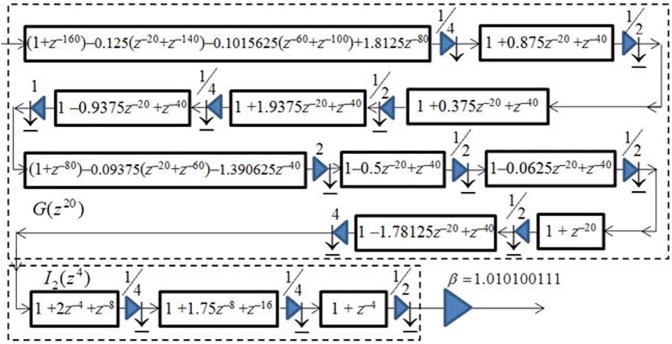
The optimally factored-cascade IFIR implementation of *U*(z) = *βG*(z^20^) *I*_2_(z^4^).

To further reduce the hardware complexity of the design (in terms of numbers of multipliers and structural adders), we used the *factoring* filter design method of [20–22] to decompose the *G*(*z*^20^) and *I*_2_(*z*^4^) filters into their optimal sequence of factors. Fig 11 shows the resulted optimally factored-cascade IFIR implementation of *U*(*z*) = *βG*(*z*^20^) *I*_2_(*z*^4^), where *β* is the post-filter-multiplier. Note that one of the main advantages of our optimally-factored cascade structure is that each stage is followed by a post-stage power-of-two multiplier and a truncation operation to properly adjust the data path word length.

The binary and Signed Powers of Two (SPT) representations of the coefficients for both the factored model filter *G*(*z*^20^) and the factored interpolator filter *I*_2_(*z*^4^) are listed in Table 1, showing that the proposed design primarily contains near-trivial coefficients. From the Table 1, we also observe that optimally-factored cascade structure has resulted in a lower hardware complexity: While the cascaded model filter *G*(*z*^20^) with order 27 only consists of 10 shift-adds and 26 structural adders, the cascaded interpolator filter *I*_2_(*z*^4^) with order 7 consists of 1 shift-adds and 5 structural adders. Therefore, our optimally-factored cascade structure of *U*(*z*) resulted in a filter with an order of 568, consisting of 13 shift-adds (or 16, if including the additional three shift-adds needed for the post-filter multiplier *β*) and 30 structural adders. This shows that the optimally-factored cascade structure of Fig 11 has indeed a lower hardware complexity than that of the double-stretch-factor IFIR implementation of *U*(*z*) from Fig 10.

**Table 1:** Quantized stages, Binary and SPT representations for the optimally factored-cascade implementation of model filter and interpolator filter of Scenario I.

Filter	Stage#	Quantized Stages	Binary Representation of Coefficients	SPT representation of coefficients
Model Filter *G*(*z*^20^)	**1**	**(1+z**^**-160**^**) -0.125 (z**^**-20**^**+z**^**-140**^**)-0.1015625(z**^**-60**^**+z**^**-100**^**) +1.8125 z**^**-80**^	**[-0.001, -0.0001101, 1.1101]**	**[–2**^**−3**^**, –2**^**−4**^**–2**^**−5**^**–2**^**−7**^ **21–2**^**−3**^**, 2**^**–**^^**4**^**]**
	**2**	**1+ 0.875 z**^**-20**^**+z**^**-40**^	**0.111**	**2**^**0**^**−2**^**−3**^
	**3**	**1+ 0.375 z**^**-20**^**+z**^**-40**^	**0.011**	**2**^**−2**^**–2**^**−3**^
	**4**	**1+ 1.9375 z**^**-20**^**+z**^**-40**^	**1.1111**	**2**^**1**^**−2**^**−4**^
	**5**	**1–0.9375 z**^**-20**^**+z**^**-40**^	**-0.1111**	**-2**^**0**^**+2**^**−4**^
	**6**	**(1+z**^**-80**^**) -0.9375 (z**^**-20**^**+z**^**-60**^**) -1.390625 z**^**-40**^	**[-0.00011, -1.011001]**	**[–2**^**−4**^**–2**^**−5**^**,– 20–2**^**−2**^**–2**^**−3**^**–2**^**−6**^**]**
	**7**	**1–0.5 z**^**-20**^**+ z**^**-40**^	**-0.1**	**-2**^**−1**^
	**8**	**1–0.0625 z**^**-20**^**+ z**^**-40**^	**-0.0001**	**-2**^**−4**^
	**9**	**1+z**^**-20**^	**None**	**None**
	**10**	**1–1.78125 z**^**-20**^**+ z**^**-40**^	**-1.11001**	**-2**^**1**^**+2**^**−2**^**–2**^**−5**^
Inter-polator Filter *I*_2_(*z*^4^)	**11**	**1+2 z**^**-4**^**+ z**^**-8**^	**10.0**	**2**
	**12**	**1+1.75 z**^**-8**^**+ z**^**-16**^	**1.11**	**2-2**^**-2**^
	**13**	**1+ z**^**-4**^	**None**	**None**

**Fig 12:**
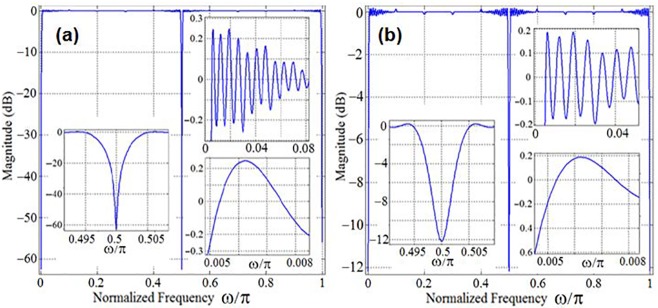
Magnitude responses of the optimally factored-cascade IFIR implementation of H(z) for two different values of post-filter-multiplier: a) β = (1.010100111)_2_; b) β = (1.0)_2_.

In addition to lower hardware complexity, the designed optimally-factored cascade structure has the capability to adjust the stopband attenuation and passband ripple of *U*(*z*) through adjustment of the post-filter-multiplier value *β*. This valuable feature of the design is highlighted in Fig 12 which shows the magnitude responses of *H*(*z*) for *β* values of (1.010100111)_2_ and (1.0)_2_.

**Fig 13:**
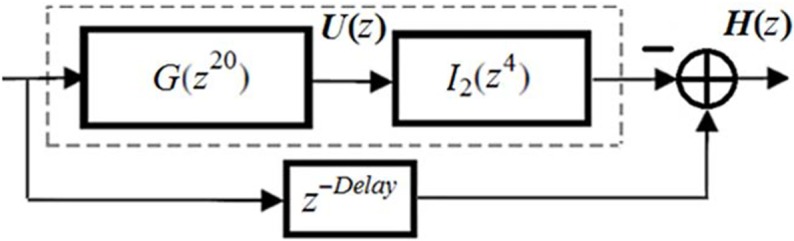
*Delay* parameter in the optimally factored-cascade IFIR implementation of *H*(*z*).

The Fig 13 *H*(*z*) implementation with the parameter *Delay*, is the extended version of Fig 10 where the ideal delay of 284 (half the effective order of the total filter) was considered. If the digital section of the ECG device includes a memory unit to record raw digital samples at the output of the analog-to-digital converter, located just before entering the digital filter, then the *Delay* parameter can be set equal to its ideal 284 value without needing any extra registers other than those used in *U*(*z*) (reusing the existing memory). Basically, the 285^th^ data sample in memory is read, and fed to the positive input of the final adder shown in Fig 13.

**Fig 14:**
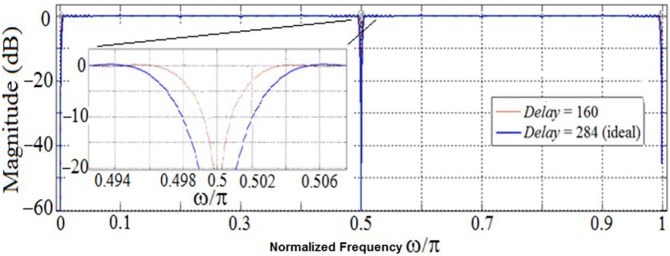
Choice of *Delay* parameter in the optimally factored-cascade IFIR implementation of *H*(*z*).

Notice that, even in the absence of such a memory unit, the proposed optimally factored-cascade IFIR structure in Fig 11 accommodates a practical choice for the *Delay* parameter, without requiring any extra register. This is illustrated in Fig 14 where the choice of *Delay* = 160 is compared with the ideal *Delay* = 284. Clearly, this *Delay* choice provides reasonable trade-offs while supporting the same level of attenuation at the stopband center frequencies (DC, 50 Hz, 100 Hz) as for the ideal scenario of *Delay* = 284. The importance of this extra choice is that the proposed cascade structures in Fig 11 inherently (at no extra cost) provides 160 registers inside its first stage. That can be used to realize the needed delay line in Fig 14. Thus, the proposed optimally-factored cascade structure of *H*(*z*) can be implemented by using 568 registers, 16 shift-adds and 31 structural adders.

Finally, using the optimal data path word length analysis [20], we found that a cascade data path of 13 bits (including the sign bit) reduces the overall stage truncation noise to a practically negligible level to meet the target filter specification.

**Fig 15:**
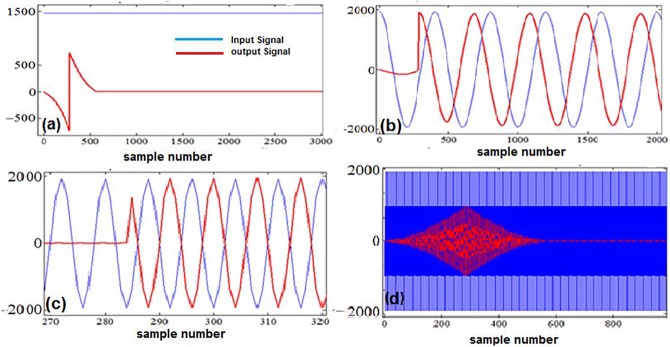
Illustration of the performance of the optimally factored-cascade IFIR implementation of H(z) for different inputs: (a) input is a DC signal; (b) input is a sinusoid at the edge of the passband; (c) input is a sinusoid in the middle of the passband; (d) input is a sinusoid at the center of the stopband.

**Fig 16:**
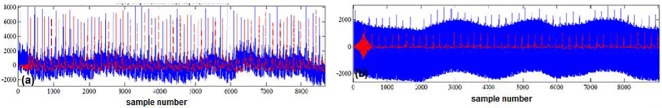
Illustration of the performance of the of the optimally factored-cascade IFIR implementation of H(z), when the input (in blue) is an ECG signal contaminated with different levels of noise: (a) The power of the noise is the same as the power of the signal; (b) The power of the noise is 100 times more than the power of the signal.

**Fig 17:**
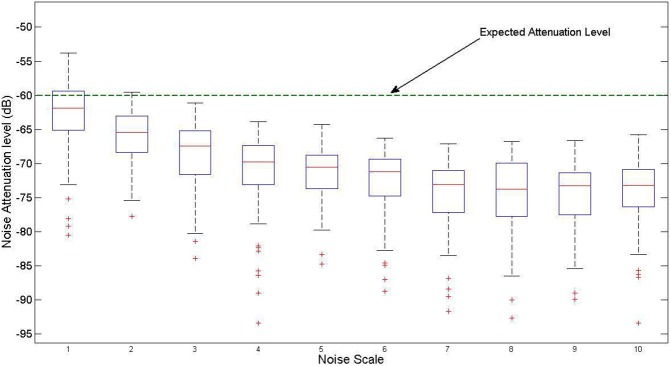
Illustration of the performance of the of the proposed filter when the input ECG data (collected with wearable sensors) is contaminated with various levels of DC and 50 Hz noise.

Fig 15, Fig 16 and Fig 17 illustrate the results of the tests that we conducted to further evaluate the performance of the designed filter. Several simulated inputs (i.e., a DC signal, a sinusoidal at the edge of the passband, a sinusoidal at the middle of the passband, and a sinusoidal at the center of the stopband) were given as the input to our filter. As Fig 15(A) shows, the designed filter successfully attenuates DC down to the desired level at the output. We also observe (Fig 15B and 15C) that the sinusoids at the edge of the passband (0.5Hz) and at the middle of passband (25 Hz) are safely passed to the outputs with no attenuation.

As Fig 15(D) shows, the 50-Hz sinusoidal input at the center of the stopband is attenuated at the output. Calculation of the average powers of the input and output signals revealed that the root mean square (RMS) of the signal has an attenuation level of 57.3 dB. This level of attenuation is achieved by using a 13-bit word length. In fact, the attenuation level for the 50-Hz sinusoidal input increases to 63.2 dB, if one uses a 14-bit word length.

To test the performance of the proposed design under extreme conditions, a real ECG signal from the publicly available MIT-BIH database [27] is resampled at 200 Hz and then contaminated with 50-Hz powerline interference and its 100-Hz harmonic. Then the proposed filter is applied to the data to suppress the noise. Fig 16(A) shows the case when the total noise power is at the same level as the incoming ECG signal power. The calculation of the output and input signals power reveals that the RMS of the output signal is approximately 3 dB lower than that of the input, as expected. This is because the designed filter *H*(*z*) removes mains-power interference associated with half the total signal power. Fig 16(B) represent a more challenging scenario, where the total power of the 50-Hz and 100-Hz noise is 100 times higher than the desired ECG signal power. We observe that, as expected, the output RMS becomes 20 dB lower than the input RMS level. These results confirm the efficacy of the proposed filter design for the noise suppression.

To complete the evaluation of the performance of the proposed design, we used the publicly available MHealth dataset [28,29]. This dataset includes more than 6 hours of body motion and vital signs recordings that were collected using wearable sensors from ten volunteers performing several physical activities (e.g. standing, sitting, climbing the stairs, cycling, jumping, jogging, running). We used 5-min ECG data segments (total of 75 segments) of this dataset for the empirical evaluation of the proposed filter. For this purpose, each data segment was resampled at 200Hz and contaminated with DC and 50 Hz noise. The power of the noise was selected as (ℕ_*S*_)^2^ times more than the power of the ECG signal, where ℕ_*S*_ was the “noise scale” changing in the range of 1 to 10. For each noise scale, the contaminated ECG data segment was fed to the proposed filter. Then the attenuation level of the noise at the output of the filter was measured in dB. Fig 17 shows the boxplots of these achieved noise attenuation levels for different input noise scales. As one expects, the filter resulted in a higher level of noise attenuation when the noise scale (the power of the contaminating noise) was larger. A t-test revealed that the achieved attenuation at each scale is significantly lower than the expected -60 dB (e.g., p-values for noise scales 1 and 2 were 10^−26^ and 10^−36^, respectively). These results confirm the effectiveness of the proposed filter design in ECG noise cancellation.

### Filter design for Scenario II

**Fig 18:**
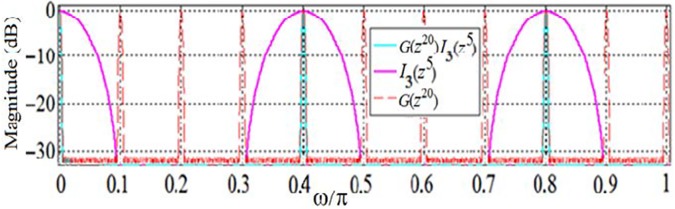
Magnitude responses of IFIR components of sub-filter *U*_*1*_(z): *G*(z^20^) and *I*_*3*_(z^5^).

Like in Scenario I, the target passband ripple (*δ*_*p*_) of Scenario II is 0.5 dB. Hence, the filter *U*_Partial_(*z*) in this scenario (a version of sub-filter *U*_*1*_(*z*) that only contains its first passband at DC) has the same IFIR components as that of the previous scenario—*G*(*z*^20^) and *I*(*z*) as shown in Fig 7. Since the magnitude response of *G*(*z*^20^) provides some of the required components of the desired magnitude response of *U*_*1*_(*z*) at DC, ±0.4π and 0.8π, we change the interpolator filter *I*(*z*) to get the desired masking illustrated in Fig 18 in solid magenta which shows the magnitude response of *I*_3_(*z*^5^), the stretched (by a factor of five) version of the new interpolator filter *I*_3_(*z*). Our analysis revealed that the interpolator filter *I*_3_(*z*) has an order of 6 (4 multipliers and 6 structural adders). Given the order of the model filter *G*(*z*) obtained in scenario I, the effective total order of *U*_*1*_(*z*) filter is (20×27) + (6×5) = 570, consisting of 18 multipliers and 33 structural adders.

**Fig 19:**
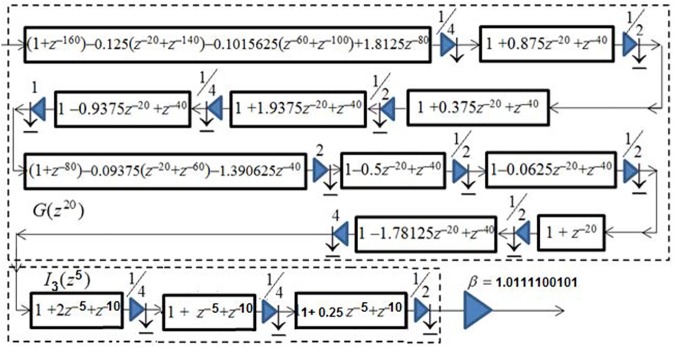
The optimally factored-cascade IFIR implementation of sub-filter *U*_*1*_(z) = *βG*(z^20^) *I*_2_(z^4^).

Fig 19 shows the resulted optimally factored-cascade IFIR implementation of the filter *U*_*1*_(*z*) = *βG*(*z*^20^) *I*_3_(*z*^5^).

The binary and SPT representations of the coefficients for both the factored model filter *G*(*z*^20^) and the factored interpolator filter *I*_3_(*z*^5^) are listed in Table 2. The optimally-factored cascade structure has resulted in a lower hardware complexity: While the cascaded model filter *G*(*z*^20^) with order 540 only consists of 10 shift-adds and 26 structural adders, the cascaded interpolator filter *I*_3_(*z*^5^) with order 30 consists of 0 shift-adds and 6 structural adders. Therefore, our optimally-factored cascade structure of *U*_*1*_(*z*) has an order of 540, and consists of 10 shift-adds (or 14, if including the additional four shift-adds needed for the post-filter multiplier *β*) and 32 structural adders.

**Table 2:** Quantized stages, Binary and SPT representations for the optimally factored-cascade implementation of model filter *G*(*z*^20^) and interpolator filter *I*_3_(*z*^5^), for sub-filter *U*_1_(*z*) of Scenario II.

Filter	Stage #	Quantized Stages	Binary Representation of Coefficients	SPT representation of coefficients
Model Filter *G*(*z*^20^)	**1**	**(1+z**^**-160**^**) -0.125 (z**^**-20**^**+z**^**-140**^**)-0.1015625(z**^**-60**^**+z**^**-100**^**) +1.8125 z**^**-80**^	**[-0.001, -0.0001101,1.1101]**	**[–2^–^^3^, –2^–^^4^–2^–^^5^–2^–^^7^, 21–2^–^^3^–2^–^^4^]**
	**2**	**1+ 0.875 z**^**-20**^**+z**^**-40**^	**0.111**	**2**^**0**^**−2**^**−3**^
	**3**	**1+ 0.375 z**^**-20**^**+z**^**-40**^	**0.011**	**2**^**−2**^**–2**^**−3**^
	**4**	**1+ 1.9375 z**^**-20**^**+z**^**-40**^	**1.1111**	**2**^**1**^**−2**^**−4**^
	**5**	**1–0.9375 z**^**-20**^**+z**^**-40**^	**-0.1111**	**-2**^**0**^**+2**^**−4**^
	**6**	**(1+z**^**-80**^**) -0.9375 (z**^**-20**^**+z**^**-60**^**) -1.390625 z**^**-40**^	**[-0.00011,-1.011001]**	**[–2^–^^4^–2^–^^5^, – 20–2^–^^2^–2^–^^3^–2^–^^6^]**
	**7**	**1–0.5 z**^**-20**^**+ z**^**-40**^	**-0.1**	**-2**^**−1**^
	**8**	**1–0.0625 z**^**-20**^**+ z**^**-40**^	**-0.0001**	**-2**^**−4**^
	**9**	**1+z**^**-20**^	**None**	**None**
	**10**	**1–1.78125 z**^**-20**^**+ z**^**-40**^	**-1.11001**	**-2**^**1**^**+2**^**−2**^**–2**^**−5**^
Inter-polator Filter *I*_3_(*z*^5^)	**11**	**1+2 z**^**-5**^**+ z**^**-10**^	**10.0**	**2**
	**12**	**1+z**^**-5**^**+ z**^**-10**^	**None**	**None**
	**13**	**1+0.25 z**^**-5**^**+ z**^**-10**^	**0.01**	**2**^**−2**^

The optimum stretch factor for the IFIR implementation of the low-pass filter *V*(*z*) was obtained as three. Using this stretch factor, we were able to realize the model filter *G*_*V*_(*z*) and interpolator filter *I*_*V*_(*z*), both with an order of 11 (each consisting of 6 multipliers and 11 structural adders). Therefore, the effective total order of filter *V*(*z*) = *G*_*V*_(*z*^*3*^) *I*_*V*_(*z*) is 3×11+11 = 44, and it consists of 12 multipliers and 22 structural adders.

**Fig 20:**
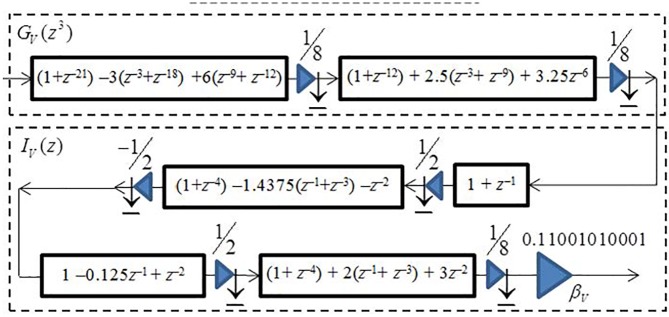
The optimally factored-cascade IFIR implementation of sub-filter *U*_*2*_(z) = *β*_*V*_*G*_*V*_(z^*3*^) *I*_*V*_(z).

Fig 20 shows the resulted optimally factored-cascade IFIR implementation of the filter *U*_*2*_(*z*) = *β*_*V*_*G*_*V*_(*z*^*3*^) *I*_*V*_(*z*).

Table 3 lists the binary and SPT representations of the coefficients for the optimally factored-cascades filters *G*_*V*_(*z*^*3*^) and *I*_*V*_(*z*). As the table shows the model filter *G*_*V*_(*z*^*3*^) with order 33 consists of 3 shift-adds and 9 structural adders, while the interpolator filter *I*_*V*_(*z*) with order 11 consists of 3 shift-adds and 11 structural adders. Therefore, our optimally-factored cascade structure of *U*_*2*_(*z*) resulted in a filter with an order of 44, consisting of 6 shift-adds (or 10, if including the additional three shift-adds needed for the post-filter multiplier *β*_*V*_) and 20 structural adders.

**Table 3:** Quantized stages, Binary and SPT representations for the optimally factored-cascade implementation of model filter *GV*(*z3*) and interpolator filter *IV*(*z*) for sub-filter *U2*(*z*) of Scenario II.

Filter	Stage #	Quantized Stages	Binary Representation of Coefficients	SPT representation of coefficients
*G_V_*(*z^3^*)	**1**	**(1+z^-21^) -3 (z^-3^+z^-18^) +6 (z^-9^+z^-12^)**	**[–11, 0, 110]**	**[2^1^+2^0^, 2^2^+2^1^]**
	**2**	**(1+z^-12^) +2.5 (z^-3^+z^-9^) +3.25 z^-6^**	**[10.10, 11.01]**	**[2^1^+2^−1^, 2^1^+2^0^+2^−2^]**
*I_V_*(*z*)	**3**	**1+z^-1^**	**None**	**None**
	**4**	**(1+z^-4^) -1.4375 (z^-1^+z^-3^) -z^-2^**	**[-1.0111, -1]**	**[-2^0^−2^−1^+2^−4^, -2^0^]**
	**5**	**1–0.125 z^-1^+ z^-2^**	**-0.001**	**-2^−3^**
	**6**	**(1+z^-4^) +2 (z^-1^+z^-3^) +3z^-2^**	**[10, 11]**	**[2^1^, 2^1^+2^0^]**

Using the obtained optimally factored-cascade IFIR implementations of the sub-filters *U*_*1*_(*z*) and *U*_*2*_(*z*), the target filter *H*(*z*) was realized using Eq (3) as

$$H(z)=[z^{-285}-U_{1}(z)][z^{-22}-V(-z)]=[z^{-285}-\beta G(z^{20})I_{3}(z^{5})][z^{-22}-\beta_{V}G_{V}(-z)I_{V}(-z)]$$ (4)

**Fig 21:**
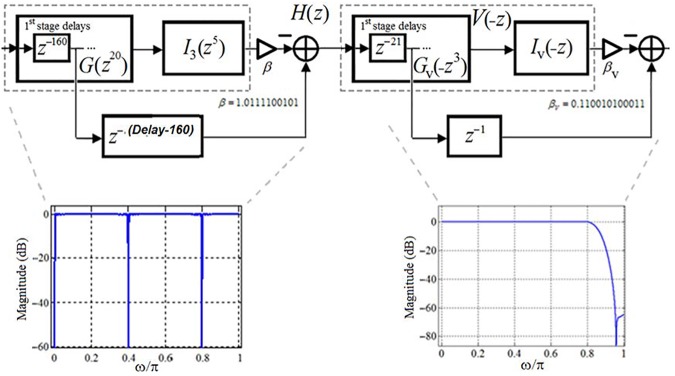
The optimally factored-cascade IFIR implementation of *H*(z).

Base on this realization, the filter *H*(*z*) has an effective order of 614, consisting of 24 shift-adds and 54 structural adders. Notice that the first stage of *V*(*z*) in Fig 20 has an order of 21, and hence can provide us with 21 of the total 22 registers that we need to realize the *z*^–22^ delay line in Eq (4), as shown in Fig 21. The first stage of *G*(*z*^20^) in Fig 19 has an order of 160, and hence can also provide us with 160 of the total 285 registers that we need to realize the *z*^–285^ delay line.

Similar to our proposed filter design for scenario I, here we employed the parameter *Delay* to enhance the robustness of the filter in terms of its total numbers of registers. Although the ideal value of the parameter *Delay* is 285 (half the effective order of sub-filter *U*_*1*_(*z*)), as our analysis from Fig 14 revealed, a *Delay* value choice of 160 provides reasonable trade-offs while supporting the same level of attenuation at the stopband center frequencies (DC, 60 Hz, 120 Hz) as for the ideal value of *Delay*. Using *Delay* = 160, we don’t need any more extra registers to realize the target filter. Thus, the proposed optimally-factored cascade structure of *H*(*z*) can be implemented by using 615 registers, 24 shift-adds and 54 structural adders.

## Discussion and conclusions

In this work, we employed a newly-developed optimally factored filter technique [20–22] combined with the optimally-stretched interpolated FIR method [23] to create a hardware-efficient ECG filter structure for two different scenarios. Our proposed designs have several beneficial features. For example, the proposed filter structures only include few shift-adds and structural adders due to the optimal factorization of the IFIR filter components. Furthermore, the proposed structures allow for the adjustment of the stopband attenuation and passband ripple of the filter, as needed, through adjustment of the value of the post-filter-multiplier *β*. Leveraging the order of the first factor (stage) filter (in the optimally factored-cascaded structure), and the parameter *Delay*, we were able to reduce the numbers of registers. The choice of *Delay* = 160 (as proposed in our design) is a reasonable choice, because both the 50 Hz and 60 Hz powerline frequencies are closely controlled by the grid. The real-time frequency of the national grid system in U.K. is available in [30], and the results of an analysis of 60 Hz main frequency accuracy are presented in [31]. Both powerline frequencies have narrow power spectral densities within ±0.2 Hz around the center frequency. Given this level of stability, the choice of *Delay* = 160 (as depicted in Fig 14) is justified as depicted in Fig 14. Furthermore, the proposed filter structures have the flexibility to easily support pipelining by inserting registers between stages [20].

Our proposed optimally-factored IFIR implementation of the ECG filter of scenario I (sampling frequency of 200 Hz, notches at DC, 50 Hz and 100 Hz) resulted in a filter with order of 568, requiring 568 registers, 16 shift-adds, and 31 structural adders. Our ECG filter design of scenario II (sampling frequency of 300 Hz, notches at DC, 60 Hz and 120 Hz) resulted in a filter with order of 614, requiring 615 registers, 24 shift-adds, and 54 structural adders. As Fig 21 shows, interestingly, our proposed design for Scenario II has the flexibility to realize the filter of Scenario I, as well. This can be achieved by simply removing the sub-filter *U*_2_(*z*) and replacing the low-order filter *I*_3_(*z*^5^) inside the first sub-filter *U*_1_(*z*) with the filter *I*_2_(*z*^4^) from Fig 10. This allows for a reconfigurable (programmable) realization of the two scenarios using one design.

To complete our study, we compared the complexity of our proposed designs to the state-of-the-art in terms of total number of full adders and flip-flops, as depicted in Table 4. Note that the authors in [10] have not provided some of the details of the FRM method including the passband ripple value, coefficients of the filter, number of full adders, and number of flipflops. Thus, we used a conservative estimate of these values for the table. As the table shows our filter design has a superior hardware efficiency to the state-of-the-art. In fact, comparing to the published filter structures, the proposed designs decrease the complexity by at least 18% in Scenario I and by at least 10% in Scenario II.

**Table 4:** Hardware complexity comparison for the ECG noise suppression filter.

Description	Method	Notch level (dB)	Register #	Data word length (bits)	Shift-add #	Structural adder #	Full adders	D flip-Flops	Total Complexity
**State of the Art Scenario I**	**Conventional parks-McClellan-Remez [24–26]**	**-60**	**≥ 560**	**12**	≥ 140*	**140**	≥ 5000*	**≥ 6720**	**≥ 11720**
	**RRS method [13]**	**-60**	**≈320 12-bit and 320 18-bit**	**12**	**≥ 2**	**7**	≈150*	**≈9600**	**≈9750**
**Proposed Filter Design Scenario I**	**Fig 10**	**-40**	**568**	**11**	**16**	**31**	**517**	**6248**	**6765**
	**Fig 10**	**-57.2**	**568**	**13**	**16**	**31**	**611**	**7384**	**7995**
	**Fig 10**	**-63.2**	**568**	**14**	**16**	**31**	**658**	**7952**	**8610**
**State of the Art Scenario II**	**Conventional parks-McClellan-Remez [24–26]**	**-60**	**≥ 594**	**12**	≥ 180*	≈174*	≥ 6000*	**≥ 7128**	**≥ 13128**
	**FRM Method [10]**	**-60**	**548**	**NA**	≥ 24**	**74**	≥ 1300**	≈7800*	**≥ 9100**
**Proposed Filter Design Scenario II**	**Fig 20**	**-60**	**615**	**12**	**24**	**54**	**936**	**7380**	**8196**

* Our estimates based on the conventional design.

** Our estimates for FRM method.

In conclusion, we presented a novel low-complexity ECG noise-suppression reconfigurable FIR filter structure using the recently-introduced optimally factored FIR filter method. Comparing to the conventional filter designs, the proposed design reduces the hardware complexity by an average of 42% (36% complexity reduction in Scenario I, and 46% complexity reduction in Scenario II). We conclude that the reduced hardware complexity of the proposed filter structure makes it a robust design for the implementation in wearable ECG and heart monitoring devices.
